# Training Executive Functions to Improve Academic Achievement: Tackling Avenues to Far Transfer

**DOI:** 10.3389/fpsyg.2021.624008

**Published:** 2021-05-24

**Authors:** Catherine Gunzenhauser, Matthias Nückles

**Affiliations:** Department of Educational Science, University of Freiburg, Freiburg, Germany

**Keywords:** executive functions, academic achievement, far transfer, cognitive training, early childhood education, self-regulation

## Abstract

The aim of training executive functions is usually to improve the ability to attain real-life goals such as academic achievement, that is, far transfer. Although many executive function trainings are successful in improving executive functions, far transfer is more difficult to achieve (cf. Diamond and Lee, 2011; Sala and Gobet, 2020). In this perspective article, we focus on the transfer of executive function training to academic performance. First, we disentangle possible sources of transfer problems. We argue that executive functions can facilitate academic performance via two specific pathways, namely learning-related behaviors and learning-related cognitions. Further, we discuss how domain-specific factors (e.g., task-specific demands and prior knowledge) may influence the successful application of executive functions to learning in this domain. Second, we discuss how the school setting can be used to enhance executive function training with approaches to facilitating far transfer to academic achievement. Specifically, we suggest that training executive functions as a means to improve academic performance is most promising in young students, for whom both behavioral and domain-specific cognitive demands of formal schooling are quite novel challenges. Furthermore, we outline that students could be supported in far transfer of trained executive functions by being informed of the specific relevance of these skills for learning-related behaviors and by having them practice executive functions under such authentic conditions. Moreover, we suggest that in order to promote ongoing effects of executive function training beyond short-term interventions, teachers should be equipped to consider the specific executive function components that might facilitate and support students’ acquisition of a particular subject matter.

## Introduction

Training of executive functions usually aims at ultimately improving real-life goal attainment via executive function gains, for instance, in the area of academic achievement (cf. Jacob and Parkinson, 2015; Diamond and Ling, 2020; Smid et al., 2020). Indeed, a large body of research documents that well-developed executive functions are associated with successful goal attainment in many areas of life (e.g., Hofmann et al., 2012; Jacob and Parkinson, 2015; Duckworth et al., 2019). Moreover, there is evidence that executive functions can be trained (e.g., Diamond and Lee, 2011; Diamond and Ling, 2020; Sala and Gobet, 2020). Thus, executive function trainings seem to be worth considering as a starting point to support children’s development. Since most children attend school, the school setting provides the opportunity to scale up trainings in order to benefit every child. However, a major challenge in executive function training is achieving a successful transfer to goal-attainment in real-life contexts (e.g., Sala and Gobet, 2020; Nesbitt and Farran, 2021). In this article, we summarize our perspective of how transfer of executive function trainings to academic goals might be achieved.

## Executive Functions: Definition and Structure, and Trainability

Executive functions are higher-order cognitive skills that allow for the top-down control and regulation of one’s own thought processes and associated actions (cf. Miyake et al., 2000; Zelazo and Müller, 2011; Karr et al., 2018). There is debate regarding the structure of executive functions as a unitary or multidimensional construct (cf. Karr et al., 2018). An influential model by Miyake et al. (2000) has distinguished three executive function components in adults, namely, working memory/updating, inhibitory control, and cognitive flexibility/attention shifting. However, factor-analytic studies tend to find a single-factor executive function model in young children, with two-factor solutions and three-factor solutions becoming more frequent as participants grow older (Karr et al., 2018). Performance in different specific executive function tasks thus seems correlated in younger children (for a discussion of possible methodological explanations such as the use of fewer tasks in studies with younger children, see Karr et al., 2018). However, there is evicence that distinct executive function components can be distinguished even in this age group. For instance, in their meta-analysis on executive function trainings for children aged 2 to 12 years, Kassai et al. (2019) found that children who received training in working memory, inhibitory control, or cognitive flexibility tended to improve in the trained components but not in the others. Thus, in the context of executive function training, we adopt the view that it is useful to distinguish executive function components even in younger children.

There are several approaches to executive function training, including cognitive training programs practicing specific executive function tasks, classroom-based curricula, and programs focusing on mindfulness or movement (for an overview, see Diamond and Ling, 2020). With regard to cognitive training, studies with both children and adults have found substantial evidence for near transfer of executive function training to performance on untrained structurally identical tasks (e.g., Jaeggi et al., 2010; Sala and Gobet, 2017, 2019, 2020; Kassai et al., 2019; Johann and Karbach, 2020). Even considering a possible publication bias favoring studies with significant effects, this is evidence for the trainability of executive functions (cf. Karbach and Kray, 2016; Diamond and Ling, 2020).

## How Are Executive Functions Important for Academic Achievement?

Although the positive association between executive functions and academic achievement is well-documented, the question whether this reflects a causal relationship is subject to debate (see Jacob and Parkinson, 2015). It has been suggested that engagement in academic activities requires children to practice executive function skills (e.g., selecting relevant information, avoiding distraction, or remembering instructions) and might therefore result in improved executive functions (cf. Schmitt et al., 2017; Peng and Kievit, 2020). For instance, Clements et al. (2020) have found that an early math curriculum contributed to improved performance on executive function assessments. However, the bulk of literature has rather focused on the opposite direction, that is, on the contribution of executive functions to academic achievement (e.g., Best et al., 2011; McClelland and Cameron, 2012; Jacob and Parkinson, 2015; Diamond et al., 2019). Given the real-life importance of academic achievement, the prospect of improving academic achievement via executive function training has high practical relevance.

### Executive Function Training: The Problem of Far Transfer

As described above, there is evidence that executive functions can be improved by executive function training (Karbach and Kray, 2016; Diamond and Ling, 2020). However, regarding far transfer of executive function training, the available evidence is much more ambiguous (cp. Pandey et al., 2018; Smithers et al., 2018; Smid et al., 2020). Far transfer means that an executive function training results in improvements in untrained executive function components, general cognitive abilities, or domain-specific academic achievement (e.g., in mathematics or literacy). With a focus on working memory training, Sala and Gobet (2019) even suggested that “the cognitive-training program of research has shown no appreciable benefits.” In early childhood education, several classroom-based programs have tried to improve academic skills through an improvement of executive function. They often combine executive function training with program elements targeting social–emotional skills and elements targeting academic skills directly. Examples include *Tools of the Mind* (e.g., Bodrova and Leong, 2009), *Head Start REDI* (e.g., Bierman et al., 2008), the *Chicago School Readiness Project* (e.g., Raver et al., 2011), and *Foundations of Learning* (Morris et al., 2013). While some studies have reported positive effects of these programs on children’s academic achievement (e.g., Raver et al., 2011; Diamond et al., 2019), others have found no significant effects (e.g., Morris et al., 2013; Nesbitt and Farran, 2021). Moreover, since classroom-based programs often target both executive functions and academic achievement, causal associations of the executive function training on academic achievement are hard to investigate (cf. Jacob and Parkinson, 2015). In their review on this matter, Jacob and Parkinson (2015) found only few methodologically sound studies allowing for conclusions (e.g., interventions that targeted executive functions but not academic achievement, random assignment, and inclusion of strong control variables such as students’ intelligence). They therefore concluded that there was no compelling evidence that improvements in executive functions result in improved academic skills (Jacob and Parkinson, 2015).

One explanation for the limited evidence for far transfer of executive function training to academic achievement might be that executive functions are simply not causally relevant to these domains. In our view, however, the lack of far transfer does not necessarily imply that executive function is useless for academic skill improvement. Instead, it could simply reflect a transfer problem, that is, a difficulty to apply newly improved executive function skills to learning assignments and academic tasks in meaningful ways. Recently, Smid et al. (2020) have made suggestions for tackling the problem of far transfer, such as gaining better insights into the mechanisms explaining the associations between training and transfer and taking into account individual differences as well as the context of transfer situations. Taking up these authors’ suggestions, we elaborate on avenues to apply executive function training in real-life academic contexts. We identify two processes relevant to academic achievement that might be boosted by well-developed executive functions: learning-related behaviors and learning-related cognitions.

### Executive Functions and Learning-Related Behaviors

It can be argued that there are some clearly defined learning-related behaviors that are likely to benefit academic achievement, such as paying attention in class, following classroom rules, and completing homework (cf. McClelland and Cameron, 2012). These behaviors are addressed in many classroom-based programs for early childhood education (e.g., Raver et al., 2011; Morris et al., 2013). As Duckworth et al. (2019) pointed out, students who have already established strong preferential habits (such as doing their homework every day after lunch) may be able to show these desirable learning-related behaviors without having to draw heavily on their executive function skills. These behaviors are then initiated automatically in response to situational clues (e.g., student finishes lunch), so that the deliberate behavior management involving executive functions is not necessary (cf. Zelazo and Müller, 2011). However, executive functions may help to establish favorable habits in new situations (e.g., a first grader needs to find ways of paying attention in class and completing her homework every day, despite having more attractive alternatives such as interacting with her classmates or watching her favorite TV shows) but become less relevant once a habit is established. Accordingly, there is evidence that executive function training can improve classroom behavior in young children (Brock et al., 2018). Furthermore, longitudinal studies have revealed evidence that learning-related behaviors mediate the association between executive functions and academic skills in children (e.g., Nesbitt et al., 2015; Sasser et al., 2015). However, this pattern of findings was not found in all studies (cf. Brock et al., 2009; Sasser et al., 2015).

### Executive Functions and Learning-Related Cognitions

#### Task-Specific Demands

Several authors have emphasized that real-life academic tasks usually do not draw on a single executive function but require an integration and interplay of executive functions (Best et al., 2011; McClelland and Cameron, 2012; Diamond, 2013). Nevertheless, some authors have tried to pin down theoretical and empirical associations between specific executive functions and specific components of mathematics or literacy skills. For instance, Bull and Lee (2014) tried to match executive functions to specific contents of the mathematics curriculum. They suggested, for instance, that learning to deal with fractions would draw heavily on inhibitory control skills (because learners would have to inhibit the idea that larger numbers represent larger quantities). Further, switching between solution strategies for non-routine tasks might require attention shifting. Another example refers to the domain of text reading: Children might draw on working memory in order to hold the content of previously read passages in mind (cf. Gerst et al., 2017). This is consistent with correlational and longitudinal studies showing that specific executive function components contribute in distinct ways to specific components of mathematics or literacy skills (e.g., Purpura et al., 2017; Cirino et al., 2019). Thus, it seems plausible that any executive function intervention targeted on improving performance should address the specific executive function skill required for the real-life academic skill that is targeted.

#### The Role of Domain-Specific Prior Knowledge

The demands on executive functions a student is faced with when working on a specific task cannot solely be explained by characteristics of the task. They also depend on the prior knowledge of the learner. The role of prior knowledge in relation to executive functions can be illustrated by taking a closer look at reading comprehension. Following Richter and Maier (2017), reading comprehension can be conceived of as a two-step model consisting of (1) a passive, automatic monitoring process and (2) a strategic, deliberate processing mode (cp. Richter and Maier, 2017). Hence, in a first step, readers routinely comprehend a text by checking “automatically” the plausibility of the written information based on their prior knowledge and beliefs. Once a comprehension problem is detected, readers may switch to a deliberate processing mode in order to resolve the comprehension problem. The second step corresponds to the deliberate control processes involving executive functions.

As a consequence, prior knowledge might have two effects with regard to the role of executive functions in successful learning. On the one hand, sufficient prior knowledge might be a precondition of applying executive functions efficiently. For instance, in order to solve a comprehension problem while reading a text, readers should be able to activate relevant, expedient ideas in their long-term memory and inhibit irrelevant or misleading ones. On the other hand, a rich and well-organized prior knowledge might reduce the demands on executive functions during the learning process because well-developed knowledge schemas will allow the reader to comprehend large parts of the written information by using the automatic processing mode (Richter and Maier, 2017). Also, well-consolidated conceptual knowledge (e.g., about fractions, cf. Bull and Lee, 2014) might reduce the intrusion of misconceptions altogether and thus reduce the necessity for inhibitory control. Thus, it seems likely that the contributions of executive functions to learning would be particularly important when the learner faces a challenge, but has sufficient prior knowledge to effectively monitor and regulate his or her learning process. Independent of the contribution of executive functions, it should be noted that early prior knowledge has been found to be a strong and consistent predictor of later achievement (e.g., Duncan et al., 2007; Ahmed et al., 2019).

## Perspective

Executive functions can be successfully improved in children (cf. Diamond and Lee, 2011), and the school context offers an ideal setting to let every child benefit from executive function training. However, a major challenge for executive function training seems to remain far transfer, for instance, its effect on academic performance (e.g., Sala and Gobet, 2020). In the following, we outline our perspective on points to consider when trying to create a best practice for school-based executive function training striving for a reconciliation of the aim for considerable effects with feasibility concerns.

### Focus on Young Children

As described above, the effective application of executive functions to a particular subject requires some prior knowledge (cf. Richter and Maier, 2017). At the same time, it might be most useful for learning when the learner cannot yet heavily rely on knowledge-based strategies as a “shortcut” to demands on executive functions (e.g., chunking information to reduce working memory load, Gobet and Simon, 1998). As students proceed though school grades, they are more and more required to build on prior domain-specific knowledge in order to master domain-specific learning goals (e.g., a student who has failed to acquire sufficient basic arithmetic skills will not be able to learn dealing with fractions just using executive functions). Thus, once students have accumulated large deficits with regard to a specific school subject, it seems less likely that they might be able to close these gaps solely by improving executive functions (cf. Duncan et al., 2007; Ahmed et al., 2019).

For these reasons, it seems advisable to focus on executive function training in young children. This recommendation is, of course, in line with efforts of a large community of researchers and practitioners focusing on the promotion of executive functions as a part of early childhood education (e.g., Bierman et al., 2008; Raver et al., 2011; Morris et al., 2013; Blair and Raver, 2015; McClelland et al., 2017). Notably, even before formal school entry, there are substantial individual differences in children’s early academic skills and executive functions (e.g., Blair and Raver, 2015). However, since curricula at the start of formal schooling do assume little prior domain-specific academic knowledge, there might be an opportunity to catch up. For instance, in early literacy instruction, every child gets the chance to learn every letter of the alphabet, despite the fact that some children might already know them (see Scammacca et al., 2020, for a summary of the mixed evidence on academic growth trajectories in the first grades). Fostering children’s executive functions at this stage might provide them with the ability to use this opportunity. In line with the well-documented positive correlation between academic achievement and executive functions, children starting formal schooling with low academic skills tend to show low executive functions as well. Notably, these children at risk for academic failure might benefit from executive function interventions even if the intervention does not provide them with excellent but merely with average executive functions. For instance, Morgan and colleagues found that children with learning-related behavior problems (Morgan et al., 2011) and very poor executive functions (Morgan et al., 2019) tended to fall increasingly behind their peers academically. Consistently, Ribner et al. (2017) reported an interaction effect between children’s preschool executive functions and mathematics skills in predicting academic skills in fifth grade. For instance, fifth-graders who had started off with a low level of mathematics skills but a high level of executive function skills were able to approximate the mathematics skills of their peers who had started off with a medium level of mathematics skills but lower executive function skills (Ribner et al., 2017).

### Identify the Specific Executive Functions Needed for Specific Target Academic Skills

Many school-based interventions targeting academic achievement through executive functions tend to provide a comprehensive, multifaceted program targeting the behavioral and emotional regulation of children (i.e., Bierman et al., 2008; Raver et al., 2011; Morris et al., 2013). This approach takes into account numerous possible factors that might facilitate academic skills. However, it makes it harder to specify how exactly a child can apply a particular executive function skill acquired through the intervention to successfully deal with the cognitive task demands when working on a specific academic task. Smid et al. (2020) suggested that research in cognitive training should analyze the specific associations between training mechanisms and the target task. For example, Jaeggi et al. (2008, 2010), suggested that their n-back working memory training task shares overlapping cognitive processes with their transfer tasks (i.e., Raven matrices). Specifically, Jaeggi et al. (2008, 2010) argued that both types of tasks require the ability to maintain a number of items or abstract relations in working memory. Hence, working memory capacity can be assumed to be an important capacity constraint that applies both to the n-back task and to *Gf*-measures such as Raven matrices. Accordingly, following the example of Jaeggi et al. (2008, 2010), executive function trainings could benefit from a cognitive task analysis (Tofel-Grehl and Feldon, 2013) that explicates in as much detail as possible which specific executive functions aid the performance in more complex learning-related behavior or cognitive tasks that the training aims to improve. The training could then focus on these executive function components. This demand may at a first glance appear as a matter of course. However, in existing cognitive training research, associations between executive function training and accumulated measures of academic achievement are frequently investigated without suggesting theory-driven and differentiated hypotheses on unique contributions of specific executive function components to specific learning-related behaviors or cognitive processes. Of course, in a laboratory setting, it might be possible to identify associations between trained skills and specific outcomes on an even more fine-grained level than an educator would be able to recognize in a real-life school context (Smid et al., 2020).

### Facilitate Far Transfer: Practice Informed Training in Real-Life Contexts

Even after identifying the specific executive function component one aims to improve, the far transfer issue remains to be tackled. A recommendation for improving far transfer of executive function training to real-life tasks can be derived from situated learning theory (Collins et al., 1989; Greeno, 1998; Bodrova and Leong, 2009). Theorists in the situated learning tradition have argued that cognitive skills need to be practiced within authentic contexts, that is, within those concrete real-life situations where the respective skill is supposed to be mastered and applied by the learner (see Renkl et al., 1996). In laboratory executive function training studies, the principle of “skill acquisition in authentic contexts” is often not considered inasmuch as executive functions are trained using tasks that have no reference to real life because they were invented by cognitive psychologists for basic research purposes.

Related to the idea of cognitive training in authentic contexts is the principle of training under varying contextual conditions (see Bodrova and Leong, 2009). As described above, several classroom-based programs in early childhood education have taken this approach (e.g., Bierman et al., 2008; Raver et al., 2011; cf. Diamond and Ling, 2020). For instance, the *Tools of the Mind* early childhood curriculum developed by Bodrova and colleagues (cp. Bodrova and Leong, 2009) contains numerous examples of how executive functions and other basic cognitive skills can be practiced under varying contextual conditions (e.g., making plans before role play or using visual reminders to practice turn-taking). Other important training principles are further inherent in these examples, these being the modeling of the focal skill by the teacher (e.g., the teacher can demonstrate how to make a plan and monitor its implementation in a multistep task, cf. Collins et al., 1989) and informed training. The latter refers to the provision of information about how, when, and why to enact a particular skill (e.g., the teacher can alert students to task characteristics that require holding information in mind, making plans, and resisting seductive irrelevant information, so-called conditional knowledge; see Paris et al., 1983). As mentioned above, effectiveness studies on *Tools of the Mind* have revealed mixed findings with regard to improvement of executive functions and academic skills. For instance, Blair and colleagues have reported positive longitudinal effects of *Tools of the Mind* on academic skills assessed with standardized achievement tests (Blair and Raver, 2014), but not on teacher reports of academic skills (Blair et al., 2018). Diamond et al. (2019) reported that *Tools of the Mind* was effective in improving reading and writing skills assessed with standardized achievement tests. Baron et al. (2017) concluded from a meta-analysis that there are small positive effects of *Tools of the Mind* on reading, self-regulation (direct assessments and ratings), and mathematics achievement, but only the effect for mathematics was statistically significant. Similarly, a recent study by Nesbitt and Farran (2021) concluded that aspects of the teacher–child interaction quality seemed to be more promising to improve student performance than the specific activities described in the *Tools of the Mind* curriculum. Despite these rather mixed outcomes, the curriculum can serve as an example of a theory-based implementation of executive function training in ecologically valid contexts.

### Include Teachers

Within a standardized intervention, only a very limited selection of specific types of tasks can be targeted. Moreover, training effects might not be sustained after the termination of the intervention (e.g., Blair et al., 2018). In order to go beyond a one-time boost and address real-life demands in every school subject, it seems promising to embed the continued scaffolding of executive functions application in the instructional context. Some examples regarding executive functions and learning-related behaviors have already been developed for classroom-based interventions (e.g., Bodrova and Leong, 2009). Moreover, teachers could be educated in conducting a cognitive task analysis as discussed above in a feasible (i.e., not too time-consuming) manner. This would allow them to focus on the demands on students’ executive functions when planning instructions and tasks (e.g., whether a task requires manipulating information in working memory, whether task switching is required, or whether multistep plans need to be made). It should be noted that classic instructional design theories such as the Elaboration Theory by Reigeluth and Stein (1983, see Reigeluth, 1999) consider a thorough analysis of the learning prerequisites for a domain principle or concept that is to be taught as an essential and routinely implemented component of effective lesson planning. For instance, one cannot learn the principle force equals mass times acceleration unless one has learned the individual concepts of mass, acceleration, and force. Similar to determining the learning prerequisites of the domain principles and concepts to be taught, teachers could learn to consider the specific executive function components that might facilitate and aid students’ acquisition of a particular subject matter. For instance, solving a mathematical word problem requires students not only to translate a situational model into a mathematical model (cf. Reusser, 1997) but also to avoid getting distracted by irrelevant information such as the “background story”, names of the characters, and so on. When aware of this executive function demand, a teacher could either treat these details as unnecessary extraneous cognitive load and try to reduce them (cf. Diamond and Ling, 2020; e.g., by providing word problems without complex and catching situational backgrounds) or use it as an opportunity for students to train strategies to deal with challenges to inhibitory control (e.g., by alerting about the possible pitfall of being misled by irrelevant details, cf. Eitel et al., 2019). We recognize that this is a demanding task in the context of heterogeneous classrooms. However, it could be considered a part of inner differentiation (e.g., diagnosing individual student’s current knowledge and needs and offering different tasks or using different teaching modalities), which is already practiced in order to account for heterogeneous performance levels and instructional needs (cf. Deunk et al., 2018). Notably, in contrast to some systematic executive function trainings (e.g., Alloway et al., 2013), such a classroom-based training of dealing with demands on executive functions in academic tasks would not systematically increase the level of difficulty or complexitiy of demands on specific executive function components. Thus, while it might contribute to the real-life aim to help students apply executive function skills to improve learning and academic achievement, it might not result in systematic improvements in cognitive tasks designed to measure specific executive function components. However, since academic skills have also been shown to predict later executive function (e.g., Fuhs et al., 2014), this is a question to be addressed in empirical research.

A second rationale to carefully include teachers to interventions targeting children’s academic skills via executive function is more distal to the application of specific executive functions to academic tasks, but should be considered nevertheless: Both successful executive function training and academic skills seem to benefit from a supportive emotional climate and social connectedness (e.g., Diamond and Ling, 2020; Nesbitt and Farran, 2021). Here, our suggestions align with approaches already taken, for instance, by the *Chicago School Readiness Project* (Raver et al., 2011) or *Foundation of Learning* (Morris et al., 2013) to support teacher’s classroom management skills as a part of an executive function intervention.

### Implications

To sum up, we suggest that considering the abovementioned principles for the training of executive functions could be a promising avenue to better bring to bear the impressive cognitive plasticity that has been demonstrated by executive function training research to real-life tasks and performance in academic disciplines. Specifically, these principles are as follows: training in authentic contexts (i.e., students’ actual learning settings) and under varying contextual conditions (e.g., classroom and homework situations) as well as modeling and informed training. In addition, fostering executive functions in early childhood and enabling teachers to integrate executive function training in everyday instruction and by seeing it as an integral part of their lesson planning may increase the scalability of such training.

## Author Contributions

CG wrote the first draft of the manuscript. MN wrote sections of the manuscript. Both authors contributed to manuscript revision, read, and approved the submitted version.

## Conflict of Interest

The authors declare that the research was conducted in the absence of any commercial or financial relationships that could be construed as a potential conflict of interest.
